# A Game of Infection – Song of Respiratory Viruses and Interferons

**DOI:** 10.3389/fcimb.2022.937460

**Published:** 2022-06-29

**Authors:** Guo Qiang Wang, Yinuo Gu, Chao Wang, Fang Wang, Alan Chen-Yu Hsu

**Affiliations:** ^1^ Department of Pathogeny Biology, College of Basic Medical Sciences, Jilin University, Changchun, China; ^2^ Signature Research Program in Emerging Infectious Diseases, Duke – National University of Singapore (NUS) Graduate Medical School, Singapore, Singapore; ^3^ School of Medicine and Public Health, The University of Newcastle, Newcastle, NSW, Australia; ^4^ Viruses, Infections/Immunity, Vaccines and Asthma, Hunter Medical Research Institute, Newcastle, NSW, Australia

**Keywords:** influenza A virus, coronavirus, coronavirus – COVID-19, virus infection, innate immunity, innate immune system

## Abstract

Humanity has experienced four major pandemics since the twentieth century, with the 1918 Spanish flu, the 2002 severe acute respiratory syndrome (SARS), the 2009 swine flu, and the 2019 coronavirus disease (COVID)-19 pandemics having the most important impact in human health. The 1918 Spanish flu caused unprecedented catastrophes in the recorded human history, with an estimated death toll between 50 – 100 million. While the 2002 SARS and 2009 swine flu pandemics caused approximately 780 and 280,000 deaths, respectively, the current COVID-19 pandemic has resulted in > 6 million deaths globally at the time of writing. COVID-19, instigated by the SARS – coronavirus-2 (SARS-CoV-2), causes unprecedented challenges in all facets of our lives, and never before brought scientists of all fields together to focus on this singular topic. While for the past 50 years research have been heavily focused on viruses themselves, we now understand that the host immune responses are just as important in determining the pathogenesis and outcomes of infection. Research in innate immune mechanisms is crucial in understanding all aspects of host antiviral programmes and the mechanisms underpinning virus-host interactions, which can be translated to the development of effective therapeutic avenues. This review summarizes what is known and what remains to be explored in the innate immune responses to influenza viruses and SARS-CoVs, and virus-host interactions in driving disease pathogenesis. This hopefully will encourage discussions and research on the unanswered questions, new paradigms, and antiviral strategies against these emerging infectious pathogens before the next pandemic occurs.

## Introduction

Influenza A viruses (IAVs) and severe acute respiratory syndrome – coronaviruses (SARS-CoVs) are both regarded as the most important respiratory infectious pathogens in human population. Their ever-changing nature constantly escapes from our current therapeutics, while emerging into novel viruses with unpredictable lethality. We have little immunity to these new viruses, and when they evolve and if they become pandemic, the consequences would be unthinkable. The 2009 H1N1 pandemic caused devastating effects, although by sheer luck the virus was not a lethal virus. The International Health Regulations Committee (World Health Organization) concluded in their 2010 report that “the world is ill-prepared to respond to a severe influenza pandemic or to any similarly global, sustained and threatening public health emergency.” (WHO, 2011). Ten years later, the unexpected appearance of the novel SARS-CoV-2 and CoV disease 2019 (COVID-19) pandemic has again emphasised our lack of preparedness and apart from costly public health measures a complete absence of effective antiviral therapeutics. Despite serious concerns of vaccine mismatch and escape mutants (Australian Government, D.o.H., 2017; Paules et al., 2018), vaccination remains the most important preventative measure against IAVs and SARS-CoV-2. Nevertheless, there is also a need to develop immune-targeting therapeutics that can be immediately deployed at the initial phase of the pandemic before vaccines become available and accessible to all nations.

Human innate immunity is an ancient immune architecture that has evolved to protect the host from microbial insults. The host pattern recognition receptors (PRRs) recognise specific genetic features common to viruses, and these factors are essential barriers to most respiratory viruses and cross-species viral transmission.

IAVs and SARS-CoVs, in agreement with the Red Queen Hypothesis, have also evolved to withstand the selective pressure from the host immune responses (Brockhurst et al., 2014). These viruses produce not only essential proteins for viral replication but also factors that effectively weaken the host antiviral responses and enhances their virulence and survival. These virus-host interactions are the primary drivers of severe diseases in the host. While rapid advances in knowledge in the host innate antiviral responses and viral infections has been achieved by fundamental discoveries, how virus-host interactions mediate dysregulated immune responses and severe diseases remain to be further defined. Understanding these mechanisms will provide important insights in the development of effective antiviral therapeutics against these infectious pathogens.

In this Review, we summarize what the host antiviral networks have taught us, how the viruses compromise our defensive responses, and current unsolved mysteries in infection and innate immunity.

### Intracellular Sentinels and the Enablers of the Antiviral Responses

IAVs and CoVs primarily infect human airway epithelial cells for viral replication. Human IAVs specifically bind with host cell surface glycoproteins carrying terminal α2,6 linked sialic acid residues, whereas avian IAVs bind to that with α2,3 linked sialic acid residues (Ito et al., 1997; Matrosovich et al., 2004). In contrast, SARS-CoVs gain cell entry by binding to angiotensin-converting enzyme (ACE)2 on airways epithelial cells (Wang Q et al., 2020; Scialo et al., 2020). IAVs and SARS-CoVs also infect alveolar type II pneumocytes and cause viral pneumonia that often requires hospitalization (Weinheimer et al., 2012; Wahl et al., 2021). Host innate immune responses initiated by these airway epithelial cells provide critical first line of defence against viral infections.

**Figure 1:**
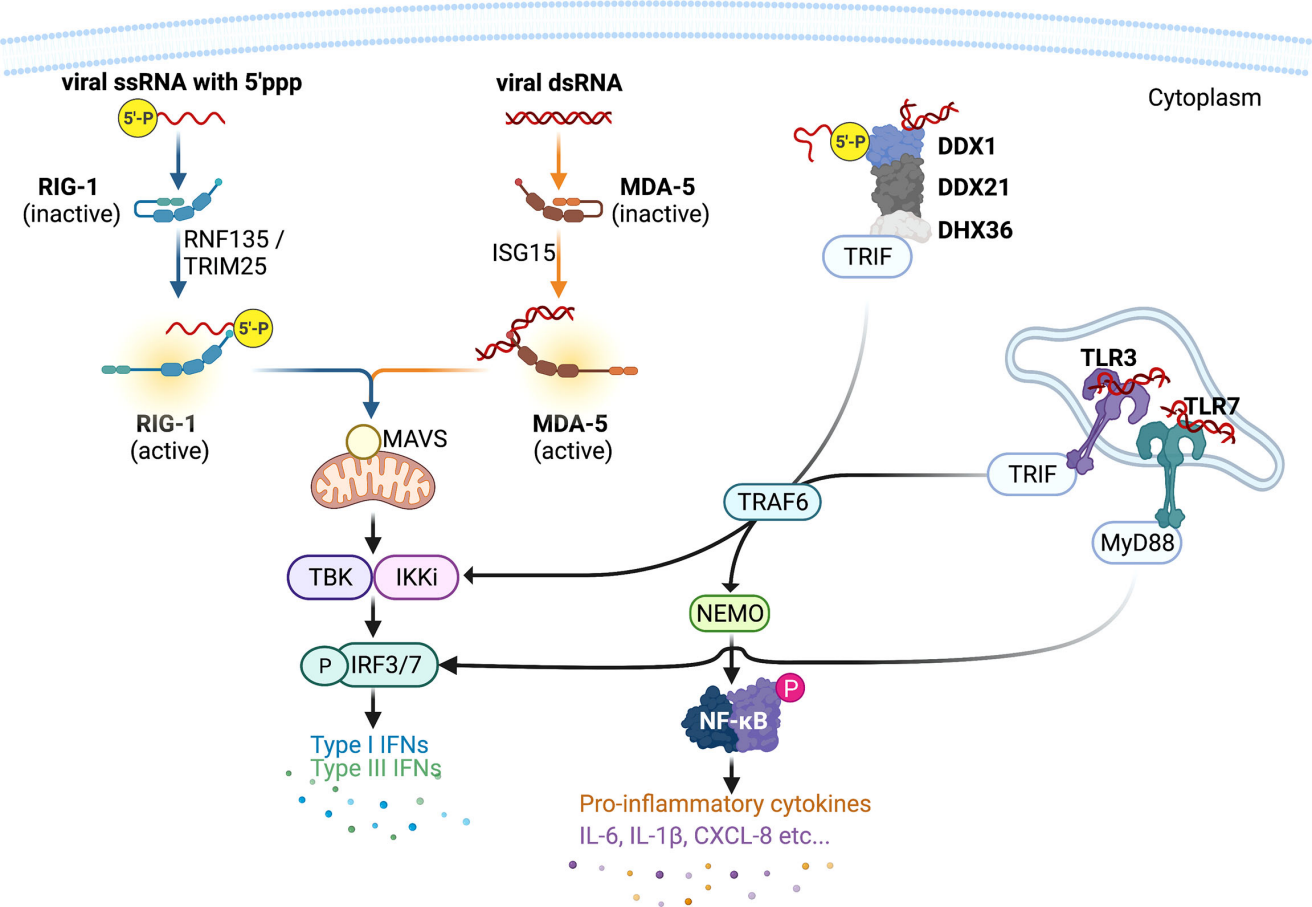
Host intracellular viral RNA recognition and innate immune signalling network. Pattern recognition receptor RIG-I recognises influenza virus ssRNAs with 5` triphosphate group, whereas MDA5, DDX1-DDX21-DHX36 complex, and TLR3 recognises viral dsRNAs. Once engaged with viral RNAs, RIG-I and MDA5 are activated by adaptor proteins RIPLET and TRIM25 and ISG15, respectively, and then initiates the production of type I and III interferons (IFNs) *via* the mitochondrial antiviral (MAVS) adaptor protein and transcription factor IFN-regulatory factor (IRF)3. DDX1-DDX21-DHX36 complex and TLR3 induces the expression of both type I and III IFNs and pro-inflammatory cytokines [interleukin (IL)-6, IL-1β, etc…] *via* the adaptor TRIF and the transcription factors IRF3/7 and nuclear factor kappa-light-chain-enhancer of activated B cells (NF-κB). This figure was created with BioRender.com.

Human innate immune system has developed a suite of intracellular PRRs to detect viral RNAs and trigger innate immune responses that restrict viral replication (**Figure 1**). There are two major classes of PRRs that facilitate the host antiviral responses, retinoic acid-inducible gene I (RIG-I)-like receptors (RLRs), endosomal Toll-like receptors (TLRs). RLRs include RIG-I and melanoma differentiation–associated gene 5 (MDA5). RIG-I specifically recognises 5′ triphosphate RNAs (5′pppRNAs) that are produced by the IAVs, whereas MDA5 and TLR3/7 binds with virus double stranded (ds)RNAs. Two recent studies independently showed that RIG-I (Thorne et al., 2021) and MDA5 (Yin et al., 2021) was crucial in inducing antiviral responses to SARS-CoV-2, however the importance of the former remains controversial as SARS-CoV-2 has not been shown to produce 5′pppRNAs. Endosomal TLR3/7 also recognise IAV RNAs and are predicted to also detect that of SARS-CoVs, and mediate both antiviral and inflammatory responses.

Once engaged with viral RNAs, RIG-I is activated by adaptor proteins RING Finger (RNF)135 (also known as RIPLET) and Tripartite Motif Containing 5 (TRIM25) (Gack et al., 2007; Hayman et al., 2019), and MDA-5 activation is dependent on interferon-inducible gene (ISG)15 (Liu et al., 2021). Activated RIG-I and MDA-5 interacts with mitochondrial antiviral signalling (MAVS) adaptor protein. MAVS forms fibrillar oligomeric complexes when activated and drives downstream antiviral cytokine production. MAVS recruits tumor-necrosis-factor (TNF)-receptor associated factor (TRAF)3, which activates TANK binding kinase (TBK1) – IκB kinase (IKK)ϵ – IKKγ complex and mediates activations of interferon (IFN) regulatory factor 3/7 (Paz et al., 2011). Activated IRFs then translocate into nucleus where they facilitate the production of antiviral proteins including type I IFNs [IFN-ɑ/-β/-κ/-ϵ (epsilon)/-ω (omega)] and type III IFNs IFN-λ1 [interleukin-29 (IL-29)], IFN-λ2 (IL-28A), IFN-λ3 (IL-28B), and IFN-λ4. The released IFNs act on the same/neighbouring cells to induce the expression of over 300 IFN-stimulated genes (ISGs) *via* the transcription factor signal transducer and activator of transcription (STAT)1. Notable ISGs that have been well characterised include protein kinase R (PKR) that inhibits viral replication and induces apoptosis (Stark et al., 1998; Fitzgerald et al., 2003; Seth et al., 2005; Hsu et al., 2012; Hsu et al., 2016), and IFN-inducible transmembrane (IFITM) 1, 2, and 3 proteins that entrap infecting virions within endosome and subsequent elimination in the endolysomes (Brass et al., 2009; Feeley et al., 2011; Compton et al., 2016).

In addition to RLRs and TLR3/, Asp-Glu-Ala-Asp (DEAD) box (DDX)1 protein is another PRR that has been shown to be important in IFN responses. Both RIG-I (aka. DDX58) and DD1 belongs to the same DEAD protein family. DDX1 forms a biomolecular condensate with DDX21 and Asp-Glu-Ala-His (DEAH) box (DHX)36 proteins (**Figure 1**) and induces type I IFNs *via* the adaptor toll-interleukin receptor (TIR)-containing adaptor molecule-1 (TICAM-1)/TIR domain-containing adaptor-induced IFN-β (TRIF) (Fuller-Pace, 2006; Zhang et al., 2011).

Endosomal TLR3 also employs TRIF as its adaptor protein and facilitates the activation of IKKɑ/β/γ complex (aka. NEMO) and nuclear factor kappa-light-chain-enhancer of activated B cells (NF-κB). NF-κB then drives the production of type I IFNs and pro-inflammatory cytokines and chemokines including interleukin (IL)-6, IL-1β, CXCL-8, and TNF-ɑ (Vallabhapurapu and Karin, 2009). These cytokines recruit immune cells such as macrophages, neutrophils, and natural killer (NK) cells to the site of infection and remove virus-infected cells (Biron et al., 1999; Mandelboim et al., 2001; Hsu et al., 2016). TLR7 and its adaptor myeloid differentiation primary response (MyD)88 mediates activation of IRF7 and subsequent transcription of type I IFNs.

IAV infection can lead to heightened inflammation (aka. cytokine storm) that promotes tissue destruction and pneumonia (Hsu et al., 2017; Scharenberg et al., 2019; Vanders et al., 2019; Gu et al., 2019). Excessive IL-6, IL-1β, and TNFα levels along with increased recruitment of immune cells in the airways contribute to epithelial damage and acute lung injury, driving the development of acute respiratory distress syndrome (ARDS), leading to severe vascular leakage and pulmonary edema (Short et al., 2014). Local inflammation can spill over into the systemic circulation, causing systemic inflammation, sepsis, and death.

Similar to IAV infection, excessive airway inflammation and epithelial damage also occurs in severe COVID-19. In addition to elevated IL-1β and TNF-ɑ productions (Sheridan et al., 1997; Kolb et al., 2001; Kimura et al., 2009; Al-Sadi et al., 2013; Kimura et al., 2013), chemokine CCL2 that is typically released by damaged cells and recruits monocytes has also been shown to be increased in COVID-19 patients (Huang et al., 2020; Li et al., 2020). These uncontrolled inflammation and tissue damage compromises the integrity of epithelial-endothelial (air-blood) barrier, leading to ARDS that is evident in severe COVID-19 (Huang et al., 2020; McGonagle et al., 2020; Mehta et al., 2020) as well as in highly pathogenic avian IAV H5N1 infection (Chotpitayasunondh et al., 2005; Tisoncik et al., 2012; Gu et al., 2019).

### Constitutively Protective or Pathogenic IFNs

#### Protective, constitutive, and Inducible IFNs

**Figure 2:**
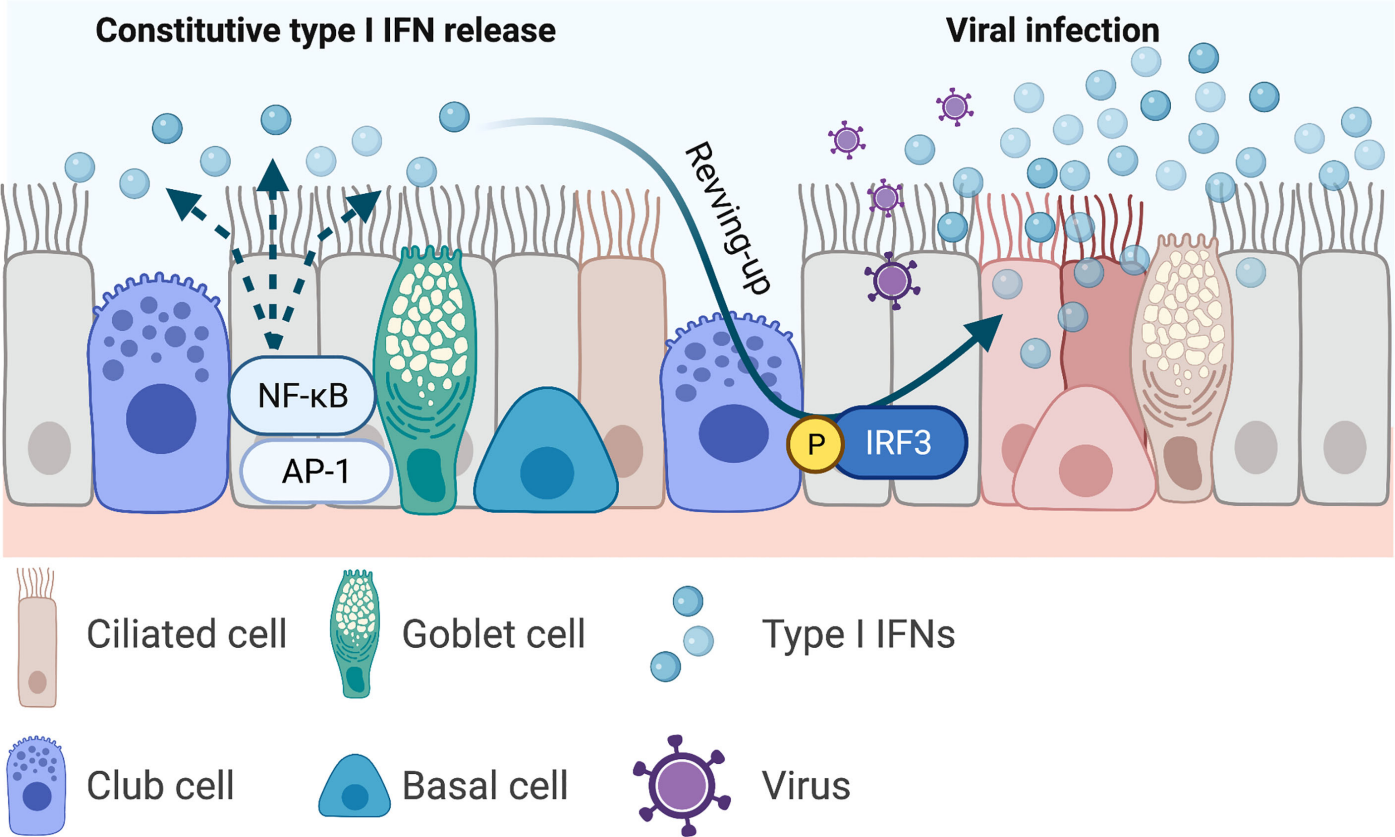
Constitutive type I interferons and the revving-up model. Airway epithelial-derived type I interferons (IFNs) are constitutively expressed and primes the local microenvironment at resting state. This priming mediates a more robust antiviral responses upon viral infection. Current evidence shows that the constitutively expressed type I IFNs are driven by the transcription factors nuclear factor kappa-light-chain-enhancer of activated B cells (NF-κB) and activating protein (AP)1. Infection induced type I IFNs are mediated by the transcription factor IFN regulatory factor (IRF)3. Increased production of type I IFNs restrict viral replication. This figure was created with BioRender.com.

The innate immune cytokines are thought to be only induced upon virus infection, reports have demonstrated that type I IFNs were constitutively expressed at low levels at resting state and primed the epithelial cells for a more robust antiviral responses to infection (**Figure 2**). This “revving-up” model has been observed with both IFN-ɑ and IFN-β in IAV and hepatitis C virus (HCV) infection (Hsu et al., 2012; Tsugawa et al., 2014), and may be driven by the constitutive expression of IRF3. This however is unlikely as IRF3 dimerization is not evident at resting state (Yoo et al., 2014; Liu et al., 2015), and IRF3 deficiency had no effect on the production of constitutive IFN-β (Hata et al., 2001). Alternative mechanisms are possibly involved in the constitutive IFN responses. Indeed, transcriptional factors NF-κB and activating protein-1 (AP-1) appears to be essential in constitutive IFN-β production (Gough et al., 2010; Balachandran and Beg, 2011; Basagoudanavar et al., 2011), as subunits of these factors (RelA and c-Jun, respectively) have been reported to bind to the IFN-β promoter region at resting state, although they have been shown to be dispensable in virus-induced IFN-β expression in airway epithelial cells. Deeper elucidation is needed to decipher the exact mechanisms by which constitutive IFNs and ISGs are maintained, particularly the interactions amongst transcriptional factors such as IRFs, NF-κB, AP-1, and other factors.

#### Pathogenic IFNs

**Figure 3:**
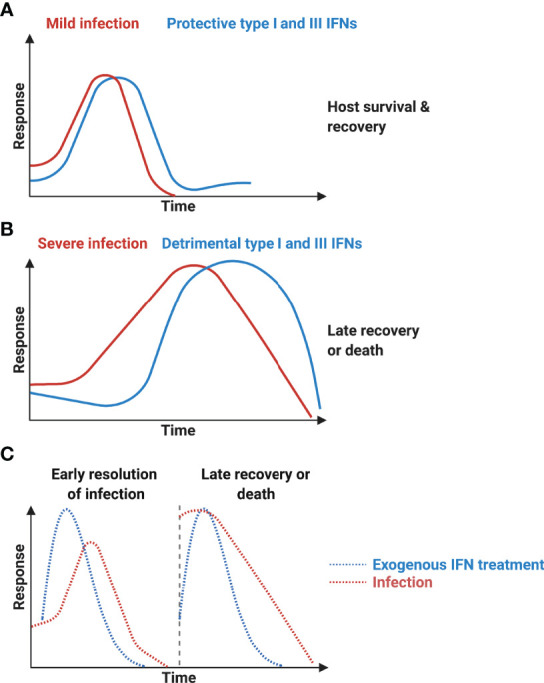
Protective interferons and interferonopathy. **(A)** Mild SARS-CoV infection is associated with early productions of type I interferons (IFNs) and resolution of viral infection. **(B)** Severe infection is accompanied with delayed but sustained type I IFN expressions and is associated with more severe diseases with increased mortality. **(C)** Early exogenous type I IFN treatment results in early resolution of COVID-19, whereas late treatment fails to show protective effects. This figure was created with BioRender.com.

Type I and III IFNs have consistently been reported to inhibit IAV viral replication in both *in vitro* (Hsu et al., 2011), *in vivo* animal models (Mordstein et al., 2008; Davidson et al., 2015), and human clinical trials (Bennett et al., 2013). However, the same protective force appears to be detrimental in severe SARS-CoV infections. Longitudinal studies revealed that while most patients with SARS-CoV infection had early IFN expression and the disease was resolved as the SARS-CoV-specific antibodies increased, those with severe diseases had initially delayed but later persistent IFN responses that were associated with reduced levels of SARS-CoV-specific antibodies and poor clinical outcomes (Cameron et al., 2007). Similarly, patients with mild-to-moderate COVID-19 also showed early IFN responses (Galani et al., 2021) (**Figure 3A**), and those with severe diseases and those deceased had increased and prolonged IFN expressions in the blood (Lucas et al., 2020) (**Figure 3B**). Interestingly, human seasonal CoV OC43 has also been shown to induce a delayed but productive viral replication with minimal type I and III IFNs as well as ISGs expressions in differentiated human primary bronchial epithelial cell model (Loo et al., 2020). The same pathological roles of IFNs have also been recapitulated in *in vivo* models. Infection with SARS-CoV or with SARS-CoV-2 showed delayed but enhanced levels of type I IFN expressions (after peak viral replication) and increased infiltration of inflammatory cytokines and macrophages, vascular leakage, and increased disease severity (Channappanavar et al., 2016; Bessiere et al., 2021). Prophylactic IFN-α treatment (1 day before infection) or early treatment post infection (1 day post inoculation) in experimental SARS-CoV-2 infection in hamster led to reduced mortality. Late IFN-α treatment (3 days post inoculation) had no protective effects (Bessiere et al., 2021) (**Figure 3C**). The protracted type I and possibly type III IFNs may be a common innate immunological feature in beta-CoV infections (including OC43 and SARS-CoVs), and this may be driven by continuous stimulation of ISG expressions. ISGs including STATs, RLRs, and IRFs further amplify type I IFN productions, which increases the recruitment and activation of immune cells into the lung. IFN-β-treated, and SARS-CoV-infected mice showed increased infiltration of macrophages in the lung compared with non-IFN treated and infected group, and IFN-β-mediated pathogenesis was reduced when monocytes were depleted (Channappanavar et al., 2016).

#### Possible Pathogenic Mechanisms by Late IFN Productions

Severe SARS-CoV-2 infection has been characterized with high levels of circulating mitochondrial (mt)DNA in the peripheral blood (Scozzi et al., 2021), strongly indicating compromised mitochondrial membrane integrity and function. mtDNAs stimulate the productions of type I IFNs expressions *via* cGAS-cGAMP-mediated STING pathway (Yang et al., 2018; Yu et al., 2020). Viral RNAs and host mtDNAs may likely trigger excessive expression of type I and III IFNs (**Table 1**).

**Table 1:** Protective early IFNs and late pathogenic IFNs in severe COVID-19.

IFN timing	Early IFNs	Late IFNs
Role	Protective	Pathogenic
Mechanisms	O Viral RNA stimulation of IFNs and ISGs O Early IFN- and ISG-mediated inhibition of virus replication O Minimal removal of virus-infected epithelial cells by macrophages and NK cells O Increased specific antibody levels	O Viral RNA and mitochondrial RNA stimulation of IFNs and ISGs O Excessive IFN- and ISG-induced cell death O ER-retained IFN-λ4, ER stress, and UPR-induced cell death

IFN production induced by viral RNAs early in infection results in successful restriction of viral replication and elimination of virus-infected cells within the airways. Late IFN inductions observed in severe COVID-19 may be the result of viral RNAs and mitochondrial (mt)DNAs released from damaged mitochondrial during infection. Enhanced expressions of IFNs, mitochondrial dysfunction and ER stress and unfolded protein response (UPR) then lead to excessive cell death.

In addition to enhanced clearance of virus-infected epithelial cells by macrophages and NK cells, exaggerated IFN levels may directly instigate sudden epithelial death. IAV infections have been shown to induce epithelial cell necroptosis (Shubina et al., 2020), and constitutive type I IFNs have recently been implicated in the induction of necroptosis *via* a key effector Mixed Lineage Kinase Domain Like Pseudokinase (MLKL) (Sarhan et al., 2019). ISGs including PKR, 2’5’A oligoadenylate synthetase (OAS), TNF-alpha related apoptosis inducing ligand (TRAIL), and galectin 9 have also been implicated in apoptosis induction (Chawla-Sarkar et al., 2003; Haw et al., 2016). While the exact ISGs and mechanisms that participate in IFN-mediated pathogenesis require further investigation, these signals enable an extensive suicidal response that clears virus-infected cells but at the expense of lung tissue integrity.

This late persistent IFN signature appears to be unique to severe COVID-19 and have not been observed with severe IAV infections (Wong et al., 2018; Turianova et al., 2019). This “interferonopathy” in COVID-19 may not be solely driven by type I IFNs. Type I and III IFNs have distinct homology and receptor utilisation but appear to induce similar ISG profiles (Kotenko et al., 2003; Sheppard et al., 2003; Prokunina-Olsson et al., 2013). While their functional differences and redundancies remain to be further defined, type I IFN-β has been reported to mediate acute ISG signatures, while type III IFN-λ induced more sustained ISG responses (Levy et al., 2011; Lin et al., 2016). IFN-λ has also been shown to be the primary effector of the antiviral defences against viral infection at the musical barrier such as the gastrointestinal tract (Pott et al., 2011; Li et al., 2019). Surprisingly, IFN-λ4, a member of the type III IFNs, was recently demonstrated to be expressed but mostly retained intracellularly in HCV and Sendai virus infection, instead of being secreted like other IFNs (Obajemu et al., 2017; Onabajo et al., 2021). Intracellular IFN-λ4 caused endoplasmic reticulum (ER) stress and the associated unfolded protein response (UPR), indicating that the translated IFN-λ4 were likely misfolded, and that the prolonged ER stress and UPR could lead to apoptotic death and tissue damage. It remains to be defined if IAV and CoV infections also cause accumulation of misfolded IFN-λ4, albeit ER stress and UPR has been demonstrated in SARS-CoV-2 infection (Koseler et al., 2020; Pathinayake et al., 2020; Bartolini et al., 2022).

The timing and magnitudes of IFN inductions, as well as the mechanisms of action of ISGs is therefore crucial in determining the molecular equipoise of protective and pathogenic IFNs. While the precise mechanisms require further investigation, it is tempting to speculate that IFN-mediated pathogenesis in COVID-19 is also attributed to viral factors.

### Counterstrike by the IAVs and SARS-CoVs

**Figure 4:**
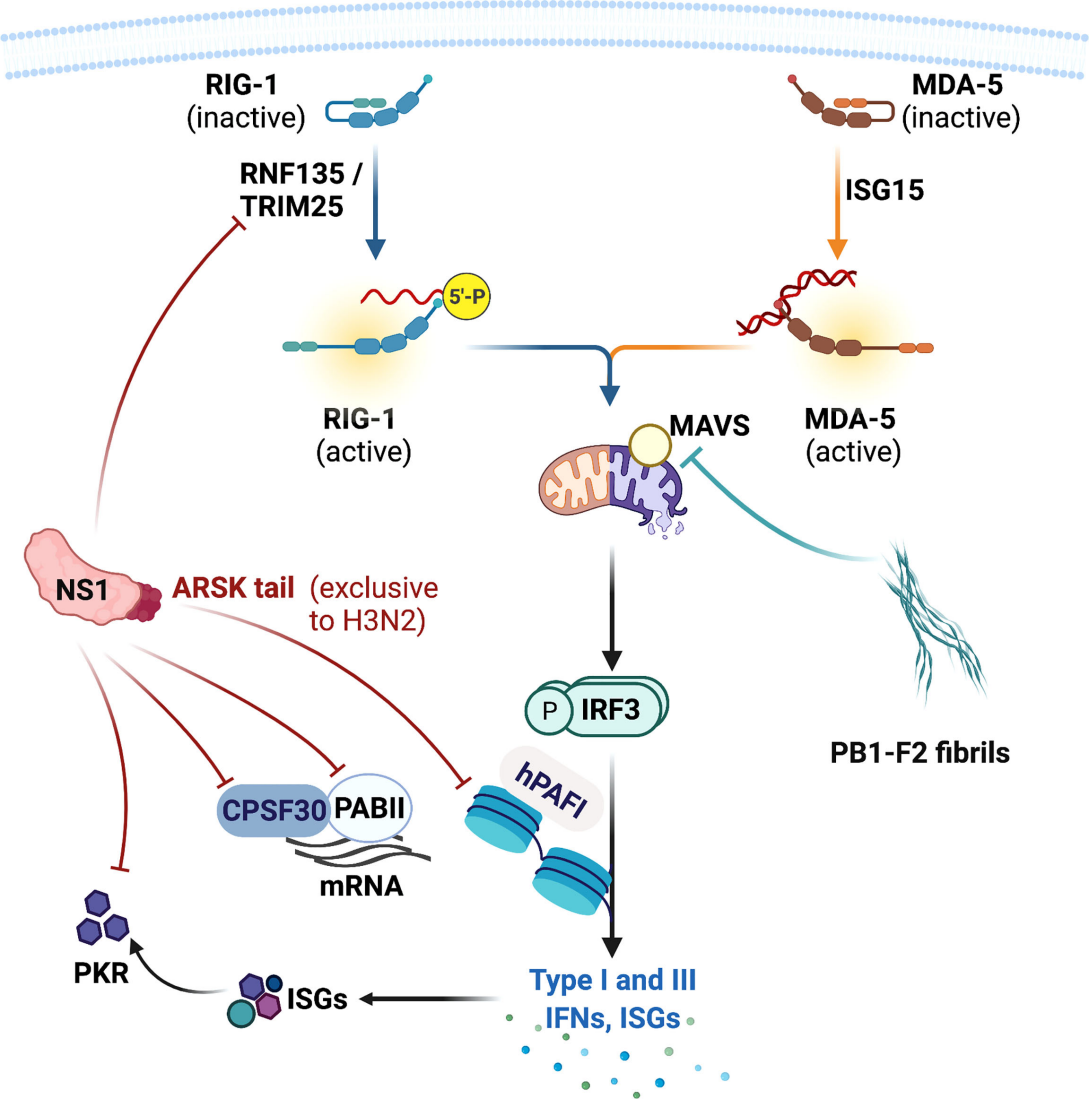
Influenza A virus virulence factors and their targets in the host. Influenza A viruses produce non-structural (NS)1 and polymerase basic 1 (PB1)-F2 proteins that inhibit host antiviral signalling and responses. NS1 protein impairs the production of type I and III IFNs by directly targeting RIG-I activators RIPLET and TRIM25, and many IFN-stimulated genes (ISGs) including protein kinase R (PKR). NS1 also inhibits host protein synthesis by targeting pre-mRNA cleavage and polyadenylation specificity factor 30 (CPSF30) and poly A binding II (PABII) proteins. NS1 also possesses a ARSK tail that inhibits human polymerase II-associated factor 1 (hPAFI) function and host protein transcription. PB1-F2 forms a fibrillar higher-molecular weight aggregate that mainly targets mitochondria and its proteins. PB1-F2 binds with mitochondrial antiviral (MAVS) protein and other mitochondrial membrane proteins, leading to inhibition of type I and III IFN productions and mitochondrial dysfunction. This also results in apoptotic cell death. This figure was created with BioRender.com.

Both IAVs and CoVs encode virulence factors that subvert host innate antiviral systems. IAV produces non-structural (NS)1 and polymerase basic (PB)1-F2 proteins that target multiple components of the IFN system (Hsu, 2018). NS1 is a small multifunctional protein that provides stealth within the cells and impairs the host antiviral responses. The RNA-binding domain of the NS1 recognises and shields viral nucleic acids from the host PRR system (Hatada and Fukuda, 1992; Qian et al., 1995; Chien et al., 2004). The non-structured effector domain stabilizes the RNA-binding domain but also interacts and interferes with host proteins including RIPLET, TRIM25, and PKR (**Figure 4**). RIPLET and TRIM25 facilitates activation of RIG-I and NS1 directly binds with these adaptors and inhibits RIG-I-mediated type I and III IFN expression (Wang et al., 2002; Bornholdt and Prasad, 2008; Rajsbaum et al., 2012). NS1 also impedes PKR from binding to viral RNAs and prevents PKR-mediated apoptosis. NS1 inhibits viral RNA recognition by PKR *via* the NS1 RNA-binding domain, and also directly interacts with PKR and suppresses PKR-mediated apoptosis through its effector domain (Bergmann et al., 2000; Dauber et al., 2006; Li et al., 2006; Min et al., 2007). Host PI3K signalling pathway is an important modulator of cellular proliferation and apoptosis. IAV has been shown to utilize this pathway as an alternative entry into cells in addition to the sialic acid residues-bearing glycoproteins (Hsu et al., 2011; Hsu et al., 2015). NS1 effector domain directly interacts with PI3K subunit p85β, further enhancing the rate of virus internalization, and this interaction also led to inhibition of PI3K-induced apoptosis (Hale et al., 2006; Shin et al., 2007; Li et al., 2008; Kedzierski et al., 2017). In addition to subverting host antiviral responses, NS1 also stalls host mRNA processing. NS1 binds with the pre-mRNA cleavage and polyadenylation specificity factor 30 (CPSF30) and poly A binding II (PABII) proteins, shutting down the host protein synthesis during infection (Nemeroff et al., 1998; Chen et al., 1999; Noah et al., 2003) (**Figure 4**).

Human H3N2 NS1 possesses a Ala-Arg-Ser-Lys (ARSK) tail at the carboxyl terminus (amino acid residues 226 – 229), which is analogous to the ART(Thr)K motif found on the lysine 4 of histone H3 (H3K4) in the host cell nucleus (Marazzi et al., 2012). Like H3K4 ARTK, NS1 ARSK tail acts as a molecular mimic that directly interacts with human polymerase II-associated factor 1 (hPAFI) and inhibits host protein synthesis (**Figure 4**). Interestingly, this ARSK tail is exclusively found in the human H3N2 and appears to be lost in other IAV subtypes, including highly pathogenic viruses H5N1, H7N9, and the pandemic H1N1 2009. The reason for this loss of a seemingly beneficial motif in other subtypes remains a mystery.

**Figure 5:**
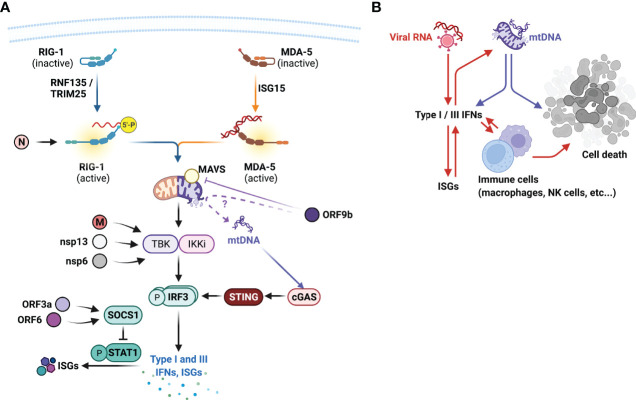
SARS-CoV virulence factors, their targets in the host and type I interferonopathies. **(A)** SARS-CoVs encode numerous viral factors that directly reduce the hosts antiviral responses. Nucleocapsid (N) protein inhibits RIG-I activation, matrix (M), non-structural protein (nsp)13, and nsp6 impairs TANK binding kinase (TBK1) activation, thus impairing the production of type I and III IFN expressions. Open-reading frame (ORF)3a and ORF6 targets suppressor of cytokine signalling (SOCS)1 that negatively regulates signal transducer and activator of transcription (STAT)1 activation. ORF9b inhibits MAVS activity, suppressing RIG-I signal transduction. Does ORF9b compromise mitochondrial membrane integrity and promote mitochondrial (mt)DNAs escape? mtDNAs further drive the production of type I IFNs *via* cytoplasmic DNA sensor cGAS and STING and contribute to the type I interferonopathies observed in severe COVID-19. **(B)** Type I interferonopathies is initially facilitated by viral RNAs that induce the expression of type I and III IFNs and IFN-stimulated genes (ISGs), which further promote the productions of these IFNs. Excessive IFNs also promote the recruitment and activation of innate immune cells including macrophages and natural killer (NK) cells that destroy virus-infected epithelial cells. Intrinsically viral proteins cause mitochondrial membrane rupture, leading to the release of mtDNAs that not only further promote IFN productions, but also cell death. This figure was created with BioRender.com.

PB1-F2 is an accessory protein translated from an alternative open reading frame of PB1. PB1-F2 promotes inflammatory cytokine storm during IAV infection (McAuley et al., 2013) and also modulate antiviral responses. PB1-F2 contains a carboxyl-terminal mitochondrial targeting sequence and directly binds with MAVS and disrupts RIG-I docking (Varga et al., 2011; Varga et al., 2012) (**Figure 5A**). PB1-F2 also targets inner and outer mitochondrial membrane transport proteins adenine nucleotide translocator 3 (ANT3) and voltage-dependent anion channel 1 (VDAC1), impairing mitochondrial membrane integrity and inducing apoptosis (Yoshizumi et al., 2014). Remarkably PB1-F2 has been shown to form a fibrillar aggregate structure that is dependent on the α-helical oligomerization domain at the carboxyl-terminus. This amyloid-like fibres have been hypothesized to form membrane pores, indicating that the PB1-F2 fibrillar structure may directly impair mitochondrial function (Chanturiya et al., 2004; Bruns et al., 2007; McAuley et al., 2013). Indeed, PB1-F2 has been demonstrated to translocate into the innate mitochondrial space *via* mitochondrial import receptor TOM40 protein (Yoshizumi et al., 2014), and avian IAV H7N9 PB1-F2 enhanced production of mitochondrial reactive oxygen species and calcium efflux, leading to mitochondrial dysfunction, inflammation and apoptosis (Pinar et al., 2017). Interestingly, full length PB1-F2 is mostly expressed by the highly pathogenic viruses including H5N1 and 1918 H1N1, as well as the human seasonal H3N2 virus. In contrast most seasonal H1N1 viruses express PB1-F2 without the carboxyl-terminus, which did not translocate into mitochondrial membrane (Yoshizumi et al., 2014). The truncated PB1-F2 of the 2009 pandemic H1N1 is thought to contribute to the reduced severity of the 2009 pandemic H1N1. This then raises an interesting question. Have the PB1-F2 of seasonal human IAVs lost all of its IFN antagonistic, inflammation- and death-inducing capabilities and what evolutionary advantages do this loss serve for the IAVs that adapt in humans? What does the truncated PB1-F2 do in the host cells? It is possible that PB1-F2 progressively loses the carboxyl-terminus as newly emerged viruses adapt in human, evolving into variants that replicate efficiently in human hosts without extensive apoptosis.

There also appears to be two opposing signals by the two most important IAV virulence factors, NS1 (anti-apoptotic) and PB1-F2 (pro-apoptotic). The molecular equipoise of apoptosis is currently unknown, although for human viruses the lost PB1-F2 carboxyl-terminus may indicate a more anti-apoptotic signals for the host cells. An inefficient virus kills it host, and a clever virus stays with it.

For SARS-CoVs, despite the protracted type I IFN signatures observed in severe COVID-19, SARS-CoVs express numerous molecules that inhibit host antiviral responses. Recent *in vitro* screening assays have indicated several SARS-CoV-2 factors that appeared to modulate host IFN responses, including structural N and M proteins, open reading frames (ORFs) 3a, 6, 7b, and 9b, and non-structural proteins (nsp) 1, 6, and 13 (Lei et al., 2020; Xia et al., 2020) (**Figure 5A**). Ectopically expressed SARS-CoV-2 N protein has been shown to bind and impede RIG-I-mediated IFN responses upon sendai virus infection (Chen et al., 2020). Whether this indicates SARS-CoV-2 viral RNA recognition by RIG-I require further investigation, but this demonstrates an interesting targeting choice by the N protein, and may indicate non-viral RNA binding functions of RIG-I.

M protein, nsp6 and nsp13 has been shown to directly interfere with TBK1 activation and inhibit IRF3 activation and IFN productions (Xia et al., 2020; Sui et al., 2021; Sui et al., 2022). ORF3a and ORF6 inhibits type I IFN responses by upregulating the suppressor of cytokine signalling (SOCS) 1 expression, a negative regulator of signal transducer and activator of transcription (STAT)1 activation (Lei et al., 2020; Wang et al., 2021) (**Figure 5A**). ORF9b has been shown to localize to the mitochondria and impair type I IFN productions *via* its interactions with MAVS and TOM70 (Shi et al., 2014; Bojkova et al., 2020; Jiang et al., 2020). This may thus explain the presence of high circulating mtDNAs in those with severe COVID-19 (Scozzi et al., 2021) and COVID-19 interferonopathies (**Figure 5A**).

It is remarkable for SARS-CoVs to encode multiple IFN-antagonistic factors, could this, along with higher levels of initial exposure to virus and viral replication, explain the delayed type I IFN and type III IFN responses in those with severe COVID-19? Expression of these viral antagonistic factors in the early phase of infection restricts early IFN responses and maximize viral replication, whilst eliciting strong pro-inflammatory responses in the airways. High levels of viral RNAs and mtDNAs from damaged mitochondria then continuously drive excessive production of IFNs, which potentially become self-sustained through positive feed-back loops (**Figure 5B**). Integrated spatial and temporal transcriptomic, proteomic, and interactomic analyses of viral and host proteins will thus provide critical insights in the dynamic expressions and interactions of viral proteins and host antiviral responses, and to reveal optimal intervention strategies.

## Concluding Perspectives

The arms race between the host and the virus dictates the outcomes of the infection. While advanced genetic screening technologies have accelerated the discovery of host intrinsic restriction factors involved in host antiviral immunity and viral infection, their detailed molecular mechanisms of actions require further investigation. This includes constitutive IFN revving-up system and how they are produced and maintained at the mucosal sites and the transcription mechanisms involved. Are they constitutively expressed at low levels and released or are they stored within the cells such as IFN-λ4 in the ER? Despite the shared Jak-STAT pathway activated by type I and III IFNs, how are they differentially and transcriptionally tuned and whether they induce unique ISG signatures also deserve further clarification. These are fundamental but underexplored areas in innate antiviral immunity, identifying the diversity and functional insights into IFN and ISG biology will provide a wealth of advances in conserved and novel mechanisms of protection from viral pathogens.

Furthermore, there are many important but unanswered questions central to the inception of interferonopathies. This includes the identities of the stimulating moieties (viral RNAs, self-DNAs (mtDNAs), and/or other unidentified molecules) that induce exaggerated type I and perhaps type III IFNs, their signalling kinetics and specific ISG repertoires and precise mechanisms that control viral infection or drive interferonopathies.

The continuous viral mutations give the viruses a huge evolutionary advantage in subverting host innate antiviral systems for survival. IAVs and SARS-CoVs virulence factors strategically compromise key PRR and IFN signalling transduction, host gene transcription, as well as ISG effector functions. As more intrinsic antiviral and cell biological factors are continually being characterised, more of which have been shown to be targeted by viral virulence factors. It is critical that we understand the full spectrum of interacting partners of the IAV and CoV virulence factors and the mutations that render the viruses more infectious or virulent. This may contribute to the surveillance of not just emerging viruses but also their virulence potentials in human.

As we head into the third year of the COVID-19 pandemic and eased restrictions, SARS-CoV-2 is likely to become epidemic and co-circulate with seasonal IAVs in the community. Several studies have reported incidences of IAV and SARS-CoV-2 co-infection amongst those recovered from severe COVID-19 and in those deceased (Wang D et al., 2020; Khodamoradi et al., 2020; Konala et al., 2020; Wu et al., 2020; Cuadrado-Payan et al., 2020; Khorramdelazad et al., 2021). Whether and how co-infection leads to worsened clinical outcomes need to be elucidated, although a recent study showed IAV and SARS-CoV-2 co-infection was significantly associated with increased risk of death (Swets et al., 2022). More importantly, IAV infection has also been shown to increase angiotensin-converting enzyme (ACE)2 expression, the host surface receptor required for SARS-CoV-2 entry into host cells, which may lead to enhanced diseases (Schweitzer et al., 2021). Is SARS-influenza or “flurona” winter coming or is it here already?

It is also important to understand how inborn error of type I IFN signalling impacts overall antiviral responses in severe viral infections and immune-targeted therapeutics such as inhaled IFN-β (Djukanović et al., 2014). In particular, autosomal-recessive deficiencies in *IRF7* and *IFNAR1* and autosomal-dominant deficiencies in *TLR3*, *TBK1*, *IRF3*, *IRF7*, *IFNAR1* and *IFNAR2* genes have been found in a small percentage of patients with severe influenza and COVID-19 (Ciancanelli et al., 2015; Lim et al., 2019; Thomsen et al., 2019). This indicates that not all would benefit from IFN treatment and alternative immune-targeting therapeutic options need to be developed. Regardless, emerging respiratory infectious viruses will always pose a threat to human and our way of life, we need to continually invest in and support research in virology, immunology, and therapeutic strategies before the next pandemic occurs.

In summary, there has been a major explosion of knowledge in PRRs and IFNs, and this will provide important understandings in critical antiviral elements in controlling viral infection. We look forward to further exciting fundamental discoveries and translational research, and how these fields might develop into potential therapeutics against emerging respiratory infectious viruses in the future.

## Author Contributions

A-YH conceptualized and wrote the manuscript with GW, YG, CW, and FW. All authors contributed to the article and approved the submitted version.

## Conflict of Interest

The authors declare that the research was conducted in the absence of any commercial or financial relationships that could be construed as a potential conflict of interest.

## Publisher’s Note

All claims expressed in this article are solely those of the authors and do not necessarily represent those of their affiliated organizations, or those of the publisher, the editors and the reviewers. Any product that may be evaluated in this article, or claim that may be made by its manufacturer, is not guaranteed or endorsed by the publisher.

## References

[B1] Al-Sadi R. Guo S. Ye D. Dokladny K. Alhmoud T. Ereifej L.. (2013). Mechanism of IL-1beta Modulation of Intestinal Epithelial Barrier Involves P38 Kinase and Activating Transcription Factor-2 Activation. J. Immunol. 190 (12), 6596–6606. doi: 10.4049/jimmunol.1201876 23656735 PMC3677168

[B2] Australian Government, D.o.H (2017). Australian Influenza Surveillance Report and Activity Updates. (Victoria, Australia: D.o. Health)

[B3] Balachandran S. Beg A. A. (2011). Defining Emerging Roles for NF-kappaB in Antivirus Responses: Revisiting the Interferon-Beta Enhanceosome Paradigm. PLoS Pathog. 7 (10), e1002165. doi: 10.1371/journal.ppat.1002165 22022260 PMC3192840

[B4] Bartolini D. Stabile A. M. Vacca C. Pistilli A. Rende M. Gioiello A.. (2022). Endoplasmic Reticulum Stress and NF-kB Activation in SARS-CoV-2 Infected Cells and Their Response to Antiviral Therapy. IUBMB Life 74 (1), 93–100. doi: 10.1002/iub.2537 34390301 PMC8426894

[B5] Basagoudanavar S. H. Thapa R. J. Nogusa S. Wang J. Beg A. A. Balachandran S. (2011). Distinct Roles for the NF-Kappa B RelA Subunit During Antiviral Innate Immune Responses. J. Virol. 85 (6), 2599–610. doi: 10.1128/JVI.02213-10 21209118 PMC3067974

[B6] Bennett A. L. Smith D. W. Cummins M. J. Jacoby P. A. Cummins J. M. Beilharz M. W. (2013). Low-Dose Oral Interferon Alpha as Prophylaxis Against Viral Respiratory Illness: A Double-Blind, Parallel Controlled Trial During an Influenza Pandemic Year. Influenza Other Respir. Viruses 7 (5), 854–862. doi: 10.1111/irv.12094 23398960 PMC5781220

[B7] Bergmann M. Garcia-Sastre A. Carnero E. Pehamberger H. Wolff K. Palese P.. (2000). Influenza Virus NS1 Protein Counteracts PKR-Mediated Inhibition of Replication. J. Virol. 74 (13), 6203–6. doi: 10.1128/JVI.74.13.6203-6206.2000 10846107 PMC112122

[B8] Bessiere P. Wasniewski M. Picard-Meyer E. Servat A. Figueroa T. Foret-Lucas C.. (2021). Intranasal Type I Interferon Treatment is Beneficial Only When Administered Before Clinical Signs Onset in the SARS-CoV-2 Hamster Model. PLoS Pathog. 17 (8), e1009427. doi: 10.1371/journal.ppat.1009427 34370799 PMC8376007

[B9] Biron C. A. Nguyen K. B. Pien G. C. Cousens L. P. Salazar-Mather T. P. (1999). Natural Killer Cells in Antiviral Defense: Function and Regulation by Innate Cytokines. Annu. Rev. Immunol. 17, 189–220. doi: 10.1146/annurev.immunol.17.1.189 10358757

[B10] Bojkova D. Klann K. Koch B. Widera M. Krause D. Ciesek S.. (2020). Proteomics of SARS-CoV-2-Infected Host Cells Reveals Therapy Targets. Nature 583 (7816), 469–472. doi: 10.1038/s41586-020-233207 32408336 PMC7616921

[B11] Bornholdt Z. A. Prasad B. V. (2008). X-Ray Structure of NS1 From a Highly Pathogenic H5N1 Influenza Virus. Nature 456 (7224), 985–8. doi: 10.1038/nature07444 18987632 PMC2798118

[B12] Brass A. L. Huang I. C. Benita Y. John S. P. Krishnan M. N. Feeley E. M.. (2009). The IFITM Proteins Mediate Cellular Resistance to Influenza A H1N1 Virus, West Nile Virus, and Dengue Virus. Cell 139 (7), 1243–54. doi: 10.1016/j.cell.2009.12.017 20064371 PMC2824905

[B13] Brockhurst M. A. Chapman T. King K. C. Mank J. E. Paterson S. Hurst G. D. (2014). Running With the Red Queen: The Role of Biotic Conflicts in Evolution. Proc. Biol. Sci. 281 (1797). doi: 10.1098/rspb.2014.1382 PMC4240979 25355473

[B14] Bruns K. Studtrucker N. Sharma A. Fossen T. Mitzner D. Eissmann A.. (2007). Structural Characterization and Oligomerization of PB1-F2, a Proapoptotic Influenza A Virus Protein. J. Biol. Chem. 282 (1), 353–363. doi: 10.1074/jbc.M606494200 17052982

[B15] Cameron M. J. Ran L. Xu L. Danesh A. Bermejo-Martin J. F. Cameron C. M.. (2007). Interferon-Mediated Immunopathological Events are Associated With Atypical Innate and Adaptive Immune Responses in Patients With Severe Acute Respiratory Syndrome. J. Virol. 81 (16), 8692–8706. doi: 10.1128/JVI.00527-07 17537853 PMC1951379

[B16] Channappanavar R. Fehr A. R. Vijay R. Mack M. Zhao J. Meyerholz D. K.. (2016). Dysregulated Type I Interferon and Inflammatory Monocyte-Macrophage Responses Cause Lethal Pneumonia in SARS-CoV-Infected Mice. Cell Host Microbe 19 (2), 181–193. doi: 10.1016/j.chom.2016.01.007 26867177 PMC4752723

[B17] Chanturiya A. N. Basanez G. Schubert U. Henklein P. Yewdell J. W. Zimmerberg J. (2004). PB1-F2, an Influenza A Virus-Encoded Proapoptotic Mitochondrial Protein, Creates Variably Sized Pores in Planar Lipid Membranes. J. Virol. 78 (12), 6304–12. doi: 10.1128/JVI.78.12.6304-6312.2004 15163724 PMC416516

[B18] Chawla-Sarkar M. Lindner D. J. Liu Y. F. Williams B. R. Sen G. C. Silverman R. H.. (2003). Apoptosis and Interferons: Role of Interferon-Stimulated Genes as Mediators of Apoptosis. Apoptosis 8 (3), 237–249. doi: 10.1023/A:1023668705040 12766484

[B19] Chen K. Xiao F. Hu D. Ge W. Tian M. Wang W.. (2020). SARS-CoV-2 Nucleocapsid Protein Interacts With RIG-I and Represses RIG-Mediated IFN-Beta Production. Viruses 13 (1), 47. doi: 10.3390/v13010047 PMC7823417 33396605

[B20] Chen Z. Li Y. Krug R. M. (1999). Influenza A Virus NS1 Protein Targets Poly(A)-Binding Protein II of the Cellular 3'-End Processing Machinery. EMBO J. 18 (8), 2273–83. doi: 10.1093/emboj/18.8.2273 10205180 PMC1171310

[B21] Chien C. Y. Xu Y. Xiao R. Aramini J. M. Sahasrabudhe P. V. Krug R. M.. (2004). Biophysical Characterization of the Complex Between Double-Stranded RNA and the N-Terminal Domain of the NS1 Protein From Influenza A Virus: Evidence for a Novel RNA-Binding Mode. Biochemistry 43 (7), 1950–62. doi: 10.1021/bi030176o 14967035

[B22] Chotpitayasunondh T. Ungchusak K. Hanshaoworakul W. Chunsuthiwat S. Sawanpanyalert P. Kijphati R.. (2005). Human Disease From Influenza A (H5N1), Thailand, 2004. Emerg. Infect. Dis. 11 (2), 201–9. doi: 10.3201/eid1102.041061 15752436 PMC3320461

[B23] Ciancanelli M. J. Huang S.X. Luthra P. Garner H. Itan Y. Volpi S.. (2015). Infectious Disease. Life-Threatening Influenza and Impaired Interferon Amplification in Human IRF7 Deficiency. Science 348 (6233), 448–453. doi: 10.1126/science.aaa1578 25814066 PMC4431581

[B24] Compton A. A. Roy N. Porrot F. Billet A. Casartelli N. Yount J. S.. (2016). Natural Mutations in IFITM3 Modulate Post-Translational Regulation and Toggle Antiviral Specificity. EMBO Rep. 17 (11), 1657–1671. doi: 10.15252/embr.201642771 27601221 PMC5090704

[B25] Cuadrado-Payan E. Montagud-Marrahi E. Torres-Elorza M. Bodro M. Blasco M. Poch E.. (2020). SARS-CoV-2 and Influenza Virus Co-Infection. Lancet 395 (10236), e84. doi: 10.1016/S0140-6736(20)31052-7 32423586 PMC7200126

[B26] Dauber B. Schneider J. Wolff T. (2006). Double-Stranded RNA Binding of Influenza B Virus Nonstructural NS1 Protein Inhibits Protein Kinase R But is Not Essential to Antagonize Production of Alpha/Beta Interferon. J. Virol. 80 (23), 11667–77. doi: 10.1128/JVI.01142-06 16987984 PMC1642593

[B27] Davidson S. Maini M. K. Wack A. (2015). Disease-Promoting Effects of Type I Interferons in Viral, Bacterial, and Coinfections. J. Interferon Cytokine Res. 35 (4), 252–264. doi: 10.1089/jir.2014.0227 25714109 PMC4389918

[B28] Djukanović R. Harrison T. Johnston S. L. Gabbay F. Wark P. Thomson N. C.. (2014). The Effect of Inhaled IFN-β on Worsening of Asthma Symptoms Caused by Viral Infections. A Randomized Trial. Am. J. Respir. Crit. Care Med. 190 (2), 145–154. doi: 10.1164/rccm.201312-2235OC 24937476 PMC4226052

[B29] Feeley E. M. Sims J. S. John S. P. Chin C. R. Pertel T. Chen L. M.. (2011). IFITM3 Inhibits Influenza A Virus Infection by Preventing Cytosolic Entry. PLoS Pathog. 7 (10), e1002337. doi: 10.1371/journal.ppat.1002337 22046135 PMC3203188

[B30] Fitzgerald K. A. McWhirter S. M. Faia K. L. Rowe D. C. Latz E. Golenbock D. T.. (2003). IKKepsilon and TBK1 are Essential Components of the IRF3 Signaling Pathway. Nat. Immunol. 4 (5), 491–6. doi: 10.1038/ni921 12692549

[B31] Fuller-Pace F. V. (2006). DExD/H Box RNA Helicases: Multifunctional Proteins With Important Roles in Transcriptional Regulation. Nucleic Acids Res. 34 (15), 4206–15. doi: 10.1093/nar/gkl460 16935882 PMC1616952

[B32] Gack M. U. Shin Y. C. Joo C. H. Urano T. Liang C. Sun L.. (2007). TRIM25 RING-Finger E3 Ubiquitin Ligase is Essential for RIG-I-Mediated Antiviral Activity. Nature 446 (7138), 916–20. doi: 10.1038/nature05732 17392790

[B33] Galani I. E. Rovina N. Lampropoulou V. Triantafyllia V. Manioudaki M. Pavlos E.. (2021). Untuned Antiviral Immunity in COVID-19 Revealed by Temporal Type I/III Interferon Patterns and Flu Comparison. Nat. Immunol. 22 (1), 32–40. doi: 10.1038/s41590-020-00840-x 33277638

[B34] Gough D. J. Messina N. L. Hii L. Gould J. A. Sabapathy K. Robertson A. P.. (2010). Functional Crosstalk Between Type I and II Interferon Through the Regulated Expression of STAT1. PLoS Biol. 8 (4), e1000361. doi: 10.1371/journal.pbio.1000361 20436908 PMC2860501

[B35] Gu Y. Hsu A. C. Pang Z. Pan H. Zuo X. Wang G.. (2019). Role of the Innate Cytokine Storm Induced by the Influenza A Virus. Viral Immunol. 32 (6), 244–51. doi: 10.1089/vim.2019.0032 31188076

[B36] Hale B. G. Jackson D. Chen Y. H. Lamb R. A. Randall R. E. (2006). Influenza A Virus NS1 Protein Binds P85beta and Activates Phosphatidylinositol-3-Kinase Signaling. Proc. Natl. Acad. Sci. U.S.A. 103 (38), 14194–9. doi: 10.1073/pnas.0606109103 16963558 PMC1599933

[B37] Hatada E. Fukuda R. (1992). Binding of Influenza A Virus NS1 Protein to dsRNA *In Vitro* . J. Gen. Virol. 73 (Pt 12), 3325–29. doi: 10.1099/0022-1317-73-12-3325 1469370

[B38] Hata N. Sato M. Takaoka A. Asagiri M. Tanaka N. Taniguchi T.. (2001). Constitutive IFN-Alpha/Beta Signal for Efficient IFN-Alpha/Beta Gene Induction by Virus. Biochem. Biophys. Res. Commun. 285 (2), 518–525. doi: 10.1006/bbrc.2001.5159 11444873

[B39] Haw T. J. Starkey M. R. Nair P. M. Pavlidis S. Liu G. Nguyen D. H.. (2016). A Pathogenic Role for Tumor Necrosis Factor-Related Apoptosis-Inducing Ligand in Chronic Obstructive Pulmonary Disease. Mucosal Immunol. 9 (4), 859–72. doi: 10.1038/mi.2015.111 26555706

[B40] Hayman T. J. Hsu A. C. Kolesnik T. B. Dagley L. F. Willemsen J. Tate M. D.. (2019). RIPLET, and Not TRIM25, is Required for Endogenous RIG-I-Dependent Antiviral Responses. Immunol. Cell Biol. 97 (9), 840–52. doi: 10.1111/imcb.12284 31335993

[B41] Hsu A. C. (2018). Influenza Virus: A Master Tactician in Innate Immune Evasion and Novel Therapeutic Interventions. Front. Immunol. 9 (743), 743. doi: 10.3389/fimmu.2018.00743 29755452 PMC5932403

[B42] Hsu A. C. Y. Barr I. Hansbro P. M. Wark P. A. (2011). Human Influenza Is More Effective Than Avian Influenza at Antiviral Suppression in Airway Cells. Am. J. Respir. Cell Mol. Biol. 44 (6), 906–913. doi: 10.1165/rcmb.2010-0157OC 20705938 PMC3135850

[B43] Hsu A. C. Parsons K. Barr I. Lowther S. Middleton D. Hansbro P. M.. (2012). Critical Role of Constitutive Type I Interferon Response in Bronchial Epithelial Cell to Influenza Infection. PloS One 7 (3), e32947. doi: 10.1371/journal.pone.0032947 22396801 PMC3292582

[B44] Hsu A. C. Starkey M. R. Hanish I. Parsons K. Haw T. J. Howland L. J.. (2015). Targeting PI3K-P110alpha Suppresses Influenza Virus Infection in Chronic Obstructive Pulmonary Disease. Am. J. Respir. Crit. Care Med. 191 (9), 1012–1023. doi: 10.1164/rccm.201501-0188OC 25751541

[B45] Hsu A. C. Parsons K. Moheimani F. Knight D. A. Hansbro P. M. Fujita T.. (2016). Impaired Antiviral Stress Granule and IFN-Beta Enhanceosome Formation Enhances Susceptibility to Influenza Infection in Chronic Obstructive Pulmonary Disease Epithelium. Am. J. Respir. Cell Mol. Biol. 55 (1), 117–127. doi: 10.1165/rcmb.2015-0306OC 26807508

[B46] Hsu A. C. Dua K. Starkey M. R. Haw T. J. Nair P. M. Nichol K.. (2017). MicroRNA-125a and -B Inhibit A20 and MAVS to Promote Inflammation and Impair Antiviral Response in COPD. JCI Insight 2 (7), e90443. doi: 10.1172/jci.insight.90443 28405612 PMC5374076

[B47] Huang C. Wang Y. Li X. Ren L. Zhao J. Hu Y.. (2020). Clinical Features of Patients Infected With 2019 Novel Coronavirus in Wuhan, China. Lancet 395 (10223), 497–506. doi: 10.1016/S0140-6736(20)30183-5 31986264 PMC7159299

[B48] Ito T. Suzuki Y. Mitnaul L. Vines A. Kida H. Kawaoka Y. (1997). Receptor Specificity of Influenza A Viruses Correlates With the Agglutination of Erythrocytes From Different Animal Species. Virology 227 (2), 493–499. doi: 10.1006/viro.1996.8323 9018149

[B49] Jiang H. W. Zhang H. N. Meng Q. F. Xie J. Li Y. Chen H.. (2020). SARS-CoV-2 Orf9b Suppresses Type I Interferon Responses by Targeting TOM70. Cell Mol. Immunol. 17 (9), 998–1000. doi: 10.1038/s41423-020-0514-8 32728199 PMC7387808

[B50] Kedzierski L. Tate M. D. Hsu A. C. Kolesnik T. B. Linossi E. M. Dagley L.. (2017). Suppressor of Cytokine Signaling (SOCS)5 Ameliorates Influenza Infection *via* Inhibition of EGFR Signaling. Elife 6:e20444. doi: 10.7554/eLife.20444.025 28195529 PMC5354519

[B51] Khodamoradi Z. Moghadami M. Lotfi M. (2020). Co-Infection of Coronavirus Disease 2019 and Influenza A: A Report From Iran. Arch. Iran Med. 23 (4), 239–43. doi: 10.34172/aim.2020.04 32271596

[B52] Khorramdelazad H. Kazemi M. H. Najafi A. Keykhaee M. Zolfaghari Emameh R. Falak R (2021). Immunopathological Similarities Between COVID-19 and Influenza: Investigating the Consequences of Co-Infection. Microb. Pathog. 152, 104554. doi: 10.1016/j.micpath.2020.104554 33157216 PMC7607235

[B53] Kimura K. Morita Y. Orita T. Haruta J. Takeji Y. Sonoda K. H. (2013). Protection of Human Corneal Epithelial Cells From TNF-Alpha-Induced Disruption of Barrier Function by Rebamipide. Invest. Ophthalmol. Vis. Sci. 54 (4), 2572–2760. 23482463 10.1167/iovs.12-11294

[B54] Kimura K. Teranishi S. Nishida T. (2009). Interleukin-1beta-Induced Disruption of Barrier Function in Cultured Human Corneal Epithelial Cells. Invest. Ophthalmol. Vis. Sci. 50 (2), 597–603. doi: 10.1167/iovs.08-2606 19171646

[B55] Kolb M. Margetts P. J. Anthony D. C. Pitossi F. Gauldie J. (2001). Transient Expression of IL-1beta Induces Acute Lung Injury and Chronic Repair Leading to Pulmonary Fibrosis. J. Clin. Invest. 107 (12), 1529–36. doi: 10.1172/JCI12568 11413160 PMC200196

[B56] Konala V. M. Adapa S. Gayam V. Naramala S. Daggubati S. R. Kammari C. B.. (2020). Co-Infection With Influenza A and COVID-19. Eur. J. Case Rep. Intern. Med. 7 (5), 001656. doi: 10.12890/2020_001656 32399452 PMC7213830

[B57] Koseler A. Sabirli R. Goren T. Turkcuer I. Kurt O. (2020). Endoplasmic Reticulum Stress Markers in SARS-COV-2 Infection and Pneumonia: Case-Control Study. In Vivo 34 (3 Suppl), 1645–1650. doi: 10.21873/invivo.11956 32503824 PMC8378033

[B58] Kotenko S. V. Gallagher G. Baurin V. V. Lewis-Antes A. Shen M. Shah N. K.. (2003). IFN-Lambdas Mediate Antiviral Protection Through a Distinct Class II Cytokine Receptor Complex. Nat. Immunol. 4 (1), 69–77. doi: 10.1038/ni875 12483210

[B59] Lei X. Dong X. Ma R. Wang W. Xiao X. Tian Z.. (2020). Activation and Evasion of Type I Interferon Responses by SARS-CoV-2. Nat. Commun. 11 (1), 3810. doi: 10.1038/s41467-020-17665-9 32733001 PMC7392898

[B60] Levy D. E. Marie I. J. Durbin J. E. (2011). Induction and Function of Type I and III Interferon in Response to Viral Infection. Curr. Opin. Virol. 1 (6), 476–86. doi: 10.1016/j.coviro.2011.11.001 22323926 PMC3272644

[B61] Li S. Min J. Y. Krug R. M. Sen G. C. (2006). Binding of the Influenza A Virus NS1 Protein to PKR Mediates the Inhibition of its Activation by Either PACT or Double-Stranded RNA. Virology 349 (1), 13–21. doi: 10.1016/j.virol.2006.01.005 16466763

[B62] Li Y. Anderson D. H. Liu Q. Zhou Y. (2008). Mechanism of Influenza A Virus NS1 Protein Interaction With the P85beta, But Not the P85alpha, Subunit of Phosphatidylinositol 3-Kinase (PI3K) and Up-Regulation of PI3K Activity. J. Biol. Chem. 283 (34), 23397–409. doi: 10.1074/jbc.M802737200 18534979

[B63] Li L. Xue M. Fu F. Yin L. Feng L. Liu P. (2019). IFN-Lambda 3 Mediates Antiviral Protection Against Porcine Epidemic Diarrhea Virus by Inducing a Distinct Antiviral Transcript Profile in Porcine Intestinal Epithelia. Front. Immunol. 10, 2394. doi: 10.3389/fimmu.2019.02394 31681286 PMC6811514

[B64] Li H. Liu L. Zhang D. Xu J. Dai H. Tang N.. (2020). SARS-CoV-2 and Viral Sepsis: Observations and Hypotheses. Lancet 395 (10235), 1517–20. doi: 10.1016/S0140-6736(20)30920-X 32311318 PMC7164875

[B65] Lim H. K. Huang S. X. L. Chen J. Kerner G. Gilliaux O. Bastard P.. (2019). Severe Influenza Pneumonitis in Children With Inherited TLR3 Deficiency. J. Exp. Med. 216 (9), 2038–56. doi: 10.1084/jem.20181621 31217193 PMC6719423

[B66] Lin J. D. Feng N. Sen A. Balan M. Tseng H. C. McElrath C.. (2016). Distinct Roles of Type I and Type III Interferons in Intestinal Immunity to Homologous and Heterologous Rotavirus Infections. PLoS Pathog. 12 (4), e1005600. doi: 10.1371/journal.ppat.1005600 27128797 PMC4851417

[B67] Liu S. Cai X. Wu J. Cong Q. Chen X. Li T.. (2015). Phosphorylation of Innate Immune Adaptor Proteins MAVS, STING, and TRIF Induces IRF3 Activation. Science 347 (6227), aaa2630. doi: 10.1126/science.aaa2630 25636800

[B68] Liu G. Lee J. H. Parker Z. M. Acharya D. Chiang J. J. van Gent M.. (2021). ISG15-Dependent Activation of the Sensor MDA5 is Antagonized by the SARS-CoV-2 Papain-Like Protease to Evade Host Innate Immunity. Nat. Microbiol. 6 (4), 467–478. doi: 10.1038/s41564-021-00884-1 33727702 PMC8103894

[B69] Loo S. L. Wark P. A. B. Esneau C. Nichol K. S. Hsu A. C. Bartlett N. W. (2020). Human Coronaviruses 229E and OC43 Replicate and Induce Distinct Anti-Viral Responses in Differentiated Primary Human Bronchial Epithelial Cells. Am. J. Physiol. Lung Cell Mol. Physiol. 319(6):L926–L931. doi: 10.1152/ajplung.00374.2020 32903043 PMC7758816

[B70] Lucas C. Wong P. Klein J. Castro T. B. R. Silva J. Sundaram M.. (2020). Longitudinal Analyses Reveal Immunological Misfiring in Severe COVID-19. Nature 584 (7821), 463–469. doi: 10.1038/s41586-020-2588-y 32717743 PMC7477538

[B71] Mandelboim O. Lieberman N. Lev M. Paul L. Arnon T. I. Bushkin Y.. (2001). Recognition of Haemagglutinins on Virus-Infected Cells by NKp46 Activates Lysis by Human NK Cells. Nature 409 (6823), 1055–1060. 11234016 10.1038/35059110

[B72] Marazzi I. Ho J. S. Kim J. Manicassamy B. Dewell S. Albrecht R. A.. (2012). Suppression of the Antiviral Response by an Influenza Histone Mimic. Nature 483 (7390), 428–433. doi: 10.1038/nature10892 22419161 PMC3598589

[B73] Matrosovich M. N. Matrosovich T. Y. Gray T. Roberts N. A. Klenk H. D. (2004). Human and Avian Influenza Viruses Target Different Cell Types in Cultures of Human Airway Epithelium. Proc. Natl. Acad. Sci. U.S.A. 101 (13), 4620–24. doi: 10.1073/pnas.0308001101 15070767 PMC384796

[B74] McAuley J. L. Tate M. D. MacKenzie-Kludas C. J. Pinar A. Zeng W. Stutz A.. (2013). Activation of the NLRP3 Inflammasome by IAV Virulence Protein PB1-F2 Contributes to Severe Pathophysiology and Disease. PLoS Pathog. 9 (5), e1003392. doi: 10.1371/journal.ppat.1003392 23737748 PMC3667782

[B75] McGonagle D. Sharif K. O'Regan A. Bridgewood C. (2020). The Role of Cytokines Including Interleukin-6 in COVID-19 Induced Pneumonia and Macrophage Activation Syndrome-Like Disease. Autoimmun Rev., 102537. doi: 10.1016/j.autrev.2020.102537 32251717 PMC7195002

[B76] Mehta P. McAuley D. F. Brown M. Sanchez E. Tattersall R. S. Manson J. J.. (2020). COVID-19: Consider Cytokine Storm Syndromes and Immunosuppression. Lancet 395 (10229), 1033–1034. doi: 10.1016/S0140-6736(20)30628-0 32192578 PMC7270045

[B77] Min J. Y. Li S. Sen G. C. Krug R. M. (2007). A Site on the Influenza A Virus NS1 Protein Mediates Both Inhibition of PKR Activation and Temporal Regulation of Viral RNA Synthesis. Virology 363 (1), 236–43. doi: 10.1016/j.virol.2007.01.038 17320139

[B78] Mordstein M. Kochs G. Dumoutier L. Renauld J. C. Paludan S. R. Klucher K.. (2008). Interferon-Lambda Contributes to Innate Immunity of Mice Against Influenza A Virus But Not Against Hepatotropic Viruses. PLoS Pathog. 4 (9), e1000151. doi: 10.1371/journal.ppat.1000151 18787692 PMC2522277

[B79] Nemeroff M. E. Barabino S. M. Li Y. Keller W. Krug R. M. (1998). Influenza Virus NS1 Protein Interacts With the Cellular 30 kDa Subunit of CPSF and Inhibits 3'end Formation of Cellular pre-mRNAs. Mol. Cell 1 (7), 991–1000. doi: 10.1016/S1097-2765(00)80099-4 9651582

[B80] Noah D. L. Twu K. Y. Krug R. M. (2003). Cellular Antiviral Responses Against Influenza A Virus are Countered at the Posttranscriptional Level by the Viral NS1A Protein *via* its Binding to a Cellular Protein Required for the 3' End Processing of Cellular pre-mRNAS. Virology 307 (2), 386–395. doi: 10.1016/S0042-6822(02)00127-7 12667806

[B81] Obajemu A. A. Rao N. Dilley K. A. Vargas J. M. Sheikh F. Donnelly R. P.. (2017). IFN-Lambda4 Attenuates Antiviral Responses by Enhancing Negative Regulation of IFN Signaling. J. Immunol. 199 (11), 3808–20. doi: 10.4049/jimmunol.1700807 29070670 PMC5698113

[B82] Onabajo O. O. Wang F. Lee M. H. Florez-Vargas O. Obajemu A. Tanikawa C.. (2021). Intracellular Accumulation of IFN-Lambda4 Induces ER Stress and Results in Anti-Cirrhotic But Pro-HCV Effects. Front. Immunol. 12, 692263. doi: 10.3389/fimmu.2021.692263 34497603 PMC8419317

[B83] Pathinayake P. S. Hsu A. C. Wark P. A. B. (2020). PAT in the ER for Transmembrane Protein Folding. Trends Biochem. Sci. 45 (12), 1007–8. doi: 10.1016/j.tibs.2020.10.001 33082068

[B84] Paules C. I. Sullivan S. G. Subbarao K. Fauci A. S. (2018). Chasing Seasonal Influenza - The Need for a Universal Influenza Vaccine. N Engl. J. Med. 378 (1), 7–9. doi: 10.1056/NEJMp1714916 29185857

[B85] Paz S. Vilasco M. Werden S. J. Arguello M. Joseph-Pillai D. Zhao T.. (2011). A Functional C-Terminal TRAF3-Binding Site in MAVS Participates in Positive and Negative Regulation of the IFN Antiviral Response. Cell Res. 21 (6), 895–910. doi: 10.1038/cr.2011.2 21200404 PMC3203699

[B86] Pinar A. Dowling J. K. Bitto N. J. Robertson A. A. Latz E. Stewart C. R.. (2017). PB1-F2 Peptide Derived From Avian Influenza A Virus H7N9 Induces Inflammation* via* Activation of the NLRP3 Inflammasome. J. Biol. Chem. 292 (3), 826–36. doi: 10.1074/jbc.M116.756379 27913620 PMC5247656

[B87] Pott J. Mahlakoiv T. Mordstein M. Duerr C. U. Michiels T. Stockinger S.. (2011). IFN-Lambda Determines the Intestinal Epithelial Antiviral Host Defense. Proc. Natl. Acad. Sci. U.S.A. 108 (19), 7944–9. doi: 10.1073/pnas.1100552108 21518880 PMC3093475

[B88] Prokunina-Olsson L. Muchmore B. Tang W. Pfeiffer R. M. Park H. Dickensheets H.. (2013). A Variant Upstream of IFNL3 (IL28B) Creating a New Interferon Gene IFNL4 is Associated With Impaired Clearance of Hepatitis C Virus. Nat. Genet. 45 (2), 164–171. doi: 10.1038/ng.2521 23291588 PMC3793390

[B89] Qian X. Y. Chien C. Y. Lu Y. Montelione G. T. Krug R. M. (1995). An Amino-Terminal Polypeptide Fragment of the Influenza Virus NS1 Protein Possesses Specific RNA-Binding Activity and Largely Helical Backbone Structure. RNA 1 (9), 948–56. 8548659 PMC1369343

[B90] Rajsbaum R. Albrecht R. A. Wang M. K. Maharaj N. P. Versteeg G. A. Nistal-Villan E.. (2012). Species-Specific Inhibition of RIG-I Ubiquitination and IFN Induction by the Influenza A Virus NS1 Protein. PLoS Pathog. 8 (11), e1003059. doi: 10.1371/journal.ppat.1003059 23209422 PMC3510253

[B91] Sarhan J. Liu B. C. Muendlein H. I. Weindel C. G. Smirnova I. Tang A. Y.. (2019). Constitutive Interferon Signaling Maintains Critical Threshold of MLKL Expression to License Necroptosis. Cell Death Differ 26 (2), 332–47. doi: 10.1038/s41418-018-0122-7 29786074 PMC6329789

[B92] Scharenberg M. Vangeti S. Kekalainen E. Bergman P. Al-Ameri M. Johansson N.. (2019). Influenza A Virus Infection Induces Hyperresponsiveness in Human Lung Tissue-Resident and Peripheral Blood NK Cells. Front. Immunol. 10, 1116. doi: 10.3389/fimmu.2019.01116 31156653 PMC6534051

[B93] Schweitzer K. S. Crue T. Nall J. M. Foster D. Sajuthi S. Correll K. A.. (2021). Influenza Virus Infection Increases ACE2 Expression and Shedding in Human Small Airway Epithelial Cells. Eur. Respir. J. 58 (1):2003988. doi: 10.1183/13993003.03988-2020 33419885 PMC8378143

[B94] Scialo F. Daniele A. Amato F. Pastore L. Matera M. G. Cazzola M.. (2020). ACE2: The Major Cell Entry Receptor for SARS-CoV-2. Lung 198 (6), 867–77. doi: 10.1007/s00408-020-00408-4 33170317 PMC7653219

[B95] Scozzi D. Cano M. Ma L. Zhou D. Zhu J. H. O'Halloran J. A.. (2021). Circulating Mitochondrial DNA is an Early Indicator of Severe Illness and Mortality From COVID-19. JCI Insight 6 (4):e143299. doi: 10.1172/jci.insight.143299 PMC7934921 33444289

[B96] Seth R. B. Sun L. Ea C. K. Chen Z. J. (2005). Identification and Characterization of MAVS, a Mitochondrial Antiviral Signaling Protein That Activates NF-kappaB and IRF 3. Cell 122 (5), 669–682. doi: 10.1016/j.cell.2005.08.012 16125763

[B97] Sheppard P. Kindsvogel W. Xu W. Henderson K. Schlutsmeyer S. Whitmore T. E.. (2003). IL-28, IL-29 and Their Class II Cytokine Receptor IL-28r. Nat. Immunol. 4 (1), 63–8. doi: 10.1038/ni873 12469119

[B98] Sheridan B. C. McIntyre R. C. Meldrum D. R. Fullerton D. A. (1997). Pentoxifylline Treatment Attenuates Pulmonary Vasomotor Dysfunction in Acute Lung Injury. J. Surg. Res. 71 (2), 150–4. doi: 10.1006/jsre.1997.5144 9299283

[B99] Shi C. S. Qi H. Y. Boularan C. Huang N. N. Abu-Asab M. Shelhamer J. H.. (2014). SARS-Coronavirus Open Reading Frame-9b Suppresses Innate Immunity by Targeting Mitochondria and the MAVS/TRAF3/TRAF6 Signalosome. J. Immunol. 193 (6), 3080–9. doi: 10.4049/jimmunol.1303196 25135833 PMC4179872

[B100] Shin Y. K. Li Y. Liu Q. Anderson D. H. Babiuk L. A. Zhou Y. (2007). SH3 Binding Motif 1 in Influenza A Virus NS1 Protein is Essential for PI3K/Akt Signaling Pathway Activation. J. Virol. 81 (23), 12730–9. doi: 10.1128/JVI.01427-07 17881440 PMC2169092

[B101] Short K. R. Kroeze E. Fouchier R. A. M. Kuiken T. (2014). Pathogenesis of Influenza-Induced Acute Respiratory Distress Syndrome. Lancet Infect. Dis. 14 (1), 57–69. doi: 10.1016/S1473-3099(13)70286-X 24239327

[B102] Shubina M. Tummers B. Boyd D. F. Zhang T. Yin C. Gautam A.. (2020). Necroptosis Restricts Influenza A Virus as a Stand-Alone Cell Death Mechanism. J. Exp. Med. 217 (11):e20191259. doi: 10.1084/jem.20191259 32797196 PMC7596817

[B103] Stark G. R. Kerr I. M. Williams B. R. Silverman R. H. Schreiber R. D. (1998). How Cells Respond to Interferons. Annu. Rev. Biochem. 67, 227–264. doi: 10.1146/annurev.biochem.67.1.227 9759489

[B104] Sui L. Zhao Y. Wang W. Wu P. Wang Z. Yu Y.. (2021). SARS-CoV-2 Membrane Protein Inhibits Type I Interferon Production Through Ubiquitin-Mediated Degradation of TBK1. Front. Immunol. 12, 662989. doi: 10.3389/fimmu.2021.662989 34084167 PMC8168463

[B105] Sui C. Xiao T. Zhang S. Zeng H. Zheng Y. Liu B.. (2022). SARS-CoV-2 NSP13 Inhibits Type I IFN Production by Degradation of TBK1 *via* P62-Dependent Selective Autophagy. J. Immunol. 208 (3), 753–61. doi: 10.4049/jimmunol.2100684 34996837

[B106] Swets M. C. Russell C. D. Harrison E. M. Docherty A. B. Lone N. Girvan M.. (2022). SARS-CoV-2 Co-Infection With Influenza Viruses, Respiratory Syncytial Virus, or Adenoviruses. Lancet. 399(10334), 1463–64. doi: 10.1016/S0140-6736(22)00383-X 35344735 PMC8956294

[B107] Thomsen M. M. Jorgensen S. E. Gad H. H. Storgaard M. Gjedsted J. Christiansen M.. (2019). Defective Interferon Priming and Impaired Antiviral Responses in a Patient With an IRF7 Variant and Severe Influenza. Med. Microbiol. Immunol. 208 (6), 869–76. doi: 10.1007/s00430-019-00623-8 31172279

[B108] Thorne L. G. Reuschl A. K. Zuliani-Alvarez L. Whelan M. V. X. Turner J. Noursadeghi M.. (2021). SARS-CoV-2 Sensing by RIG-I and MDA5 Links Epithelial Infection to Macrophage Inflammation. EMBO J. 40 (15), e107826. doi: 10.15252/embj.2021107826 34101213 PMC8209947

[B109] Tisoncik J. R. Korth M. J. Simmons C. P. Farrar J. Martin T. R. Katze M. G. (2012). Into the Eye of the Cytokine Storm. Microbiol. Mol. Biol. Rev. 76 (1), 16–32. doi: 10.1128/MMBR.05015-11 22390970 PMC3294426

[B110] Tsugawa Y. Kato H. Fujita T. Shimotohno K. Hijikata M. (2014). Critical Role of Interferon-α Constitutively Produced in Human Hepatocytes in Response to RNA Virus Infection. PLoS One 9 (2), e89869. doi: 10.1371/journal.pone.0089869 24587086 PMC3935935

[B111] Turianova L. Lachova V. Svetlikova D. Kostrabova A. Betakova T. (2019). Comparison of Cytokine Profiles Induced by Nonlethal and Lethal Doses of Influenza A Virus in Mice. Exp. Ther. Med. 18 (6), 4397–405. doi: 10.3892/etm.2019.8096 31777543 PMC6862669

[B112] Vallabhapurapu S. Karin M. (2009). Regulation and Function of NF-kappaB Transcription Factors in the Immune System. Annu. Rev. Immunol. 27, 693–733. doi: 10.1146/annurev.immunol.021908.132641 19302050

[B113] Vanders R. L. Hsu A. Gibson P. G. Murphy V. E. Wark P. A. B. (2019). Nasal Epithelial Cells to Assess *In Vitro* Immune Responses to Respiratory Virus Infection in Pregnant Women With Asthma. Respir. Res. 20 (1), 259. doi: 10.1186/s12931-019-1225-5 31747925 PMC6865028

[B114] Varga Z. T. Ramos I. Hai R. Schmolke M. Garcia-Sastre A. Fernandez-Sesma A.. (2011). The Influenza Virus Protein PB1-F2 Inhibits the Induction of Type I Interferon at the Level of the MAVS Adaptor Protein. PLoS Pathog. 7 (6), e1002067. doi: 10.1371/journal.ppat.1002067 21695240 PMC3111539

[B115] Varga Z. T. Grant A. Manicassamy B. Palese P. (2012). Influenza Virus Protein PB1-F2 Inhibits the Induction of Type I Interferon by Binding to MAVS and Decreasing Mitochondrial Membrane Potential. J. Virol. 86 (16), 8359–66. doi: 10.1128/JVI.01122-12 22674996 PMC3421771

[B116] Wahl A. Gralinski L. E. Johnson C. E. Yao W. Kovarova M. Dinnon K. H. 3rd. (2021). SARS-CoV-2 Infection is Effectively Treated and Prevented by EIDD-2801. Nature 591 (7850), 451–457. 33561864 10.1038/s41586-021-03312-w PMC7979515

[B117] Wang X. Basler C. F. Williams B. R. Silverman R. H. Palese P. Garcia-Sastre A. (2002). Functional Replacement of the Carboxy-Terminal Two-Thirds of the Influenza A Virus NS1 Protein With Short Heterologous Dimerization Domains. J. Virol. 76 (24), 12951–62. doi: 10.1128/JVI.76.24.12951-12962.2002 12438621 PMC136679

[B118] Wang Q. Zhang Y. Wu L. Niu S. Song C. Zhang Z.. (2020). Structural and Functional Basis of SARS-CoV-2 Entry by Using Human Ace2. Cell 181 (4), 894–904 e9. doi: 10.1016/j.cell.2020.03.045 32275855 PMC7144619

[B119] Wang D. Hu B. Hu C. Zhu F. Liu X. Zhang J.. (2020). Clinical Characteristics of 138 Hospitalized Patients With 2019 Novel Coronavirus-Infected Pneumonia in Wuhan, China. JAMA 323 (11), 1061–69. doi: 10.1001/jama.2020.1585 32031570 PMC7042881

[B120] Wang R. Yang X. Chang M. Xue Z. Wang W. Bai L.. (2021). ORF3a Protein of Severe Acute Respiratory Syndrome Coronavirus 2 Inhibits Interferon-Activated Janus Kinase/Signal Transducer and Activator of Transcription Signaling *via* Elevating Suppressor of Cytokine Signaling 1. Front. Microbiol. 12, 752597. doi: 10.3389/fmicb.2021.752597 34650546 PMC8506155

[B121] Weinheimer V. K. Becher A. Tonnies M. Holland G. Knepper J. Bauer T. T.. (2012). Influenza A Viruses Target Type II Pneumocytes in the Human Lung. J. Infect. Dis. 206 (11), 1685–94. doi: 10.1093/infdis/jis455 22829640 PMC7107318

[B122] WHO (2011). Report of the Review Committee on the Functioning of the International Health Regulations (2005) in Relation to Pandemic (H1N1) 2009 (World Health Organization).

[B123] Wong S. S. Oshansky C. M. Guo X. J. Ralston J. Wood T. Seeds R.. (2018). Severe Influenza Is Characterized by Prolonged Immune Activation: Results From the SHIVERS Cohort Study. J. Infect. Dis. 217 (2), 245–256. doi: 10.1093/infdis/jix571 29112724 PMC7335675

[B124] Wu Q. Xing Y. Shi L. Li W. Gao Y. Pan S.. (2020). Coinfection and Other Clinical Characteristics of COVID-19 in Children. Pediatrics 146 (1):e20200961. doi: 10.1542/peds.2020-0961 32376725

[B125] Xia H. Cao Z. Xie X. Zhang X. Chen J. Y. Wang H.. (2020). Evasion of Type I Interferon by SARS-CoV-2. Cell Rep. 33 (1), 108234. doi: 10.1016/j.celrep.2020.108234 32979938 PMC7501843

[B126] Yang K. Huang R. Fujihira H. Suzuki T. Yan N. (2018). N-Glycanase NGLY1 Regulates Mitochondrial Homeostasis and Inflammation Through NRF1. J. Exp. Med. 215 (10), 2600–16. doi: 10.1084/jem.20180783 30135079 PMC6170171

[B127] Yin X. Riva L. Pu Y. Martin-Sancho L. Kanamune J. Yamamoto Y.. (2021). MDA5 Governs the Innate Immune Response to SARS-CoV-2 in Lung Epithelial Cells. Cell Rep. 34 (2), 108628. doi: 10.1016/j.celrep.2020.108628 33440148 PMC7832566

[B128] Yoo J. S. Takahasi K. Ng C. S. Ouda R. Onomoto K. Yoneyama M.. (2014). DHX36 Enhances RIG-I Signaling by Facilitating PKR-Mediated Antiviral Stress Granule Formation. PLoS Pathog. 10 (3), e1004012. doi: 10.1371/journal.ppat.1004012 24651521 PMC3961341

[B129] Yoshizumi T. Ichinohe T. Sasaki O. Otera H. Kawabata S. Mihara K.. (2014). Influenza A Virus Protein PB1-F2 Translocates Into Mitochondria *via* Tom40 Channels and Impairs Innate Immunity. Nat. Commun. 5, 4713. doi: 10.1038/ncomms5713 25140902

[B130] Yu C. H. Davidson S. Harapas C. R. Hilton J. B. Mlodzianoski M. J. Laohamonthonkul P.. (2020). TDP-43 Triggers Mitochondrial DNA Release *via* mPTP to Activate cGAS/STING in ALS. Cell 183 (3), 636–649 e18. doi: 10.1016/j.cell.2020.09.020 33031745 PMC7599077

[B131] Zhang Z. Kim T. Bao M. Facchinetti V. Jung S. Y. Ghaffari A. A.. (2011). DDX1, DDX21, and DHX36 Helicases Form a Complex With the Adaptor Molecule TRIF to Sense dsRNA in Dendritic Cells. Immunity 34 (6), 866–78. doi: 10.1016/j.immuni.2011.03.027 21703541 PMC3652560

